# Incorporation of Collagen and Hyaluronic Acid to Enhance the Bioactivity of Fibrin-Based Hydrogels for Nucleus Pulposus Regeneration

**DOI:** 10.3390/jfb9030043

**Published:** 2018-07-10

**Authors:** Jennifer Gansau, Conor Timothy Buckley

**Affiliations:** 1 Trinity Centre for Bioengineering, Trinity Biomedical Sciences Institute, Trinity College Dublin, 2 Dublin, Ireland; jgansau@tcd.ie; 2 School of Engineering, Trinity College Dublin, 2 Dublin, Ireland; 3 Advanced Materials and Bioengineering Research (AMBER) Centre, Royal College of Surgeons in Ireland & Trinity College Dublin, 2 Dublin, Ireland

**Keywords:** fibrin, hydrogel, collagen, hyaluronan, intervertebral disc, nucleus pulposus, chondrocytes

## Abstract

Hydrogels, such as fibrin, offer a promising delivery vehicle to introduce cells into the intervertebral disc (IVD) to regenerate damaged disc tissue as a potential treatment for low back pain. However, fibrin lacks key extracellular matrix (ECM) components, such as collagen (Col) and hyaluronan (HA), normally found in native nucleus pulposus (NP) tissue. The overall aim of this work was to create a fibrin-based hydrogel, by incorporating Col and HA into the matrix to enhance NP-like matrix accumulation using articular chondrocytes (CC). Firstly, we assessed the effect of fibrin concentrations on hydrogel stability, and the viability and proliferation kinetics of articular chondrocytes. Secondly, we investigated the effect of incorporating Col and HA to enhance NP-like matrix accumulation, and finally, examined the influence of various HA concentrations. Results showed that increasing fibrin concentration enhanced cell viability and proliferation. Interestingly, incorporation of HA promoted sGAG accumulation and tended to suppress collagen formation at higher concentrations. Taken together, these results suggest that incorporation of ECM components can enhance the bioactivity of fibrin-based hydrogels, which may help advance the clinical potential of commercial cell and biomaterial ventures in the treatment of IVD regeneration.

## 1. Introduction

Low back pain (LBP) is one of the most prevalent pathologies worldwide [1], placing a substantial burden on healthcare resources. It occurs naturally with age, and is considered to be associated with degeneration of the intervertebral disc (IVD), which is characterised by increased cell death [2] and decreased accumulation of extracellular matrix (ECM) components, such as collagen and proteoglycans. These are the main constituents of nucleus pulposus (NP) tissue, providing its unique biomechanical properties. Early stage degeneration begins with a loss of proteoglycans and water content from the central part of the NP, with the consequential loss of the ability to absorb compressive loads. There has been significant interest in developing injectable cell-based therapies for the treatment of degenerated disc disease (DDD), with the specific aim to repopulate the NP and augment tissue repair. One such technique, known as autologous disc cell transplantation (ADCT) has been in clinical use for the treatment of degenerated IVD, with recent investigations demonstrating that re-implantation of extracted culture expanded NP cells can retard degenerative changes in humans with herniated discs [3]. However, there are associated limitations with ADCT, including cell leakage following injection into the disc [4], diminished matrix-forming capacity of culture-expanded NP cells derived from degenerated tissue [5], and the paucity of nucleus pulposus (NP) cells that can be isolated.

These challenges have motivated the exploration of various cell types, including chondrocytes [6,7] and stem cells [8,9,10], for the regeneration of disc tissues. In particular, articular-derived chondrocytes have been proposed for intervertebral disc repair, and have been investigated in several clinical trials for cartilage and disc repair with some success (e.g., IMPACT, co.don^®^ chondrospheres). However, it has been shown that when cells are directly injected into the target site, cell leakage and increased cell death due to shear forces may occur, inhibiting treatment success [11,12,13,14]. To overcome these issues, many studies have utilised natural polymer-derived hydrogel systems, such as alginate, collagen, and fibrin [15,16,17,18,19,20]. Hydrogels are highly attractive, as they can provide cellular protection during injection, enhancing cellular retention, and can also be tailored to regulate cellular bioactivity [21,22,23,24,25].

Fibrin is perhaps one of the most clinically used hydrogels, and has received particular attention as a sealant and adhesive in surgery [26,27,28]. Fibrin is a viscoelastic polymer which crosslinks after an enzymatic reaction between fibrinogen and thrombin, facilitating in situ gelation. Previous work has demonstrated successful encapsulation of cells into fibrin hydrogels with cells, exhibiting good viability and proliferation [7,19,20,29,30]. The primary components of fibrin, such as fibrinogen and thrombin, have been shown to modulate cell attachment, migration, and proliferation of stem cells and chondrocytes [29,31,32]. Chondrocytes have been shown to retain their rounded morphology in fibrin hydrogels, inhibiting dedifferentiation and promoting matrix production [33]. Also, the effect of different parameters, such as pH, fibrinogen, and salt concentration, have been shown to influence long term fibrin gel stability [19].

In addition, incorporation of extracellular matrix (ECM) constituents into fibrin gels, such as collagen and hyaluronic acid (HA), have been shown to enhance matrix deposition by chondrocytes [20,34]. Collagen is the most abundant protein in mammalian ECM, and supports tissue stability and structure. Hyaluronic acid is a non-sulphated GAG, and is highly abundant in NP tissue, where its function is to maintain tissue hydration. Both materials have been used separately in various studies [35,36,37,38], with HA demonstrating a beneficial effect on matrix synthesis and proliferation. However, the benefits of incorporating collagen appear to be dependent on cell type. For example, Colombini et al. showed that collagen-enriched fibrin hydrogels were suitable for culturing of annulus fibrosus (AF) cells, but not NP cells [20].

In the present study, we investigated fibrin hydrogels of different compositions in an attempt to identify a suitable composition to promote NP-like extracellular matrix accumulation that may be compatible with commercial ventures. The first objective explored the effects of fibrin concentration (12.5, 25, 37.5, and 50 mg/mL) on hydrogel stability and the viability and proliferation kinetics of articular chondrocytes. We next sought to enhance the bioactivity of fibrin-based hydrogels by incorporating key matrix components (Col and HA), and further investigated the effect on matrix formation by articular chondrocytes. Finally, we examined the influence of incorporating different HA concentrations (2.5 and 5 mg/mL) into fibrin-based hydrogels.

## 2. Results

### 2.1. Stage 1—Effect of Increasing Fibrinogen Concentration

#### 2.1.1. A Minimum Concentration of 25 mg/mL Fibrinogen Is Required to Maintain Construct Stability over 21 Days in Culture

**Figure 1:**
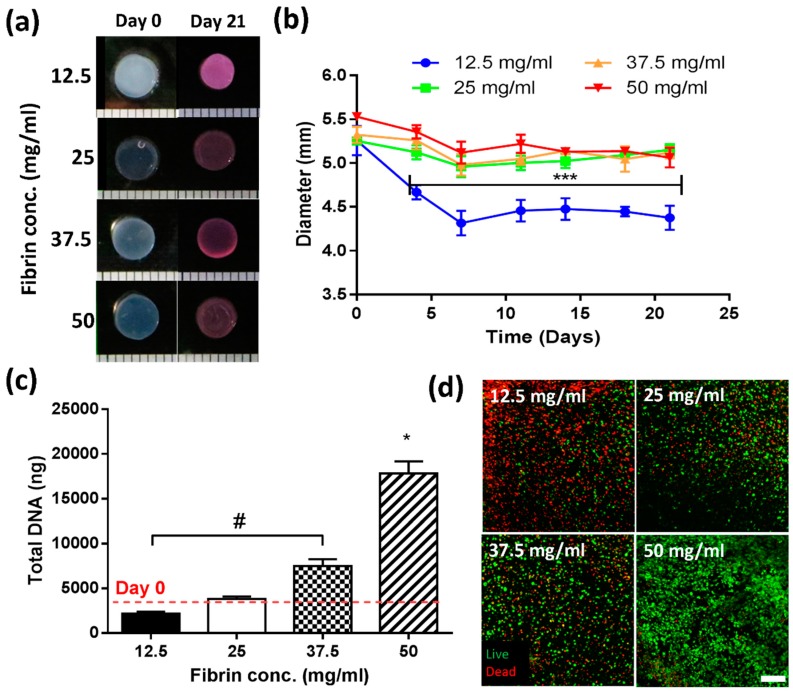
Effect of fibrinogen concentration on construct stability and cell proliferation (**a**) Macroscopic images before culture (“Day 0”, left) and after three weeks in cell culture (“Day 21”, right); (**b**) Contraction kinetics of constructs over time up to 21 days. Low concentration (12.5 mg/mL, blue) hydrogels contracted significantly over the first 7 days. *** indicates significant difference (*p* < 0.001); (**c**) Biochemical analysis of DNA content in constructs. # indicates significant difference between 12.5 and 37.5 mg/mL (*p* < 0.01) and * indicates a significant difference compared to all other groups (*p* < 0.001); (**d**) Live/dead analysis showing a high degree of cell death for the lowest concentration (12.5 mg/mL) and increased cell viability in the highest concentrations (50 mg/mL). Scale bar = 200 μm.

At the initiation of culture, all constructs possessed an average diameter of 5.46 mm (±0.19) (Figure 1a,b). After four days in culture, constructs with the lowest fibrinogen concentration (12.5 mg/mL) underwent contraction (~27%, 3.99 mm ± 0.14), whereas all other groups appeared to remain relatively stable. By day 21, the lowest concentration (12.5 mg/mL) had contracted by ~35%, with all other groups (25, 37.5, 50 mg/mL) exhibiting ~8% contraction relative to day 0.

#### 2.1.2. Increasing Fibrinogen Concentration Enhances Cell Proliferation

Fibrinogen concentration was observed to significantly influence cell proliferation (Figure 1c,d). For 12.5 mg/mL gels, DNA content was observed to decrease relative to day 0. At 25 mg/mL, no significant change was observed with time. However, for 37.5 mg/mL, a two-fold increase in DNA content was observed, which was enhanced further in 50 mg/mL gels (5-fold increase). Biochemical data was confirmed through live/dead staining (Figure 1d), where dead cells are represented in red, and viable cells in green. Significant cell death occurred at the lowest (12.5 mg/mL) concentration (top left image), with some cell death also observed for the two middle concentrations (25 and 37.5 mg/mL). By contrast, for 50 mg/mL gels, a high number of viable cells were noted (bottom right image).

### 2.2. Stage 2—Incorporation of ECM into Fibrin-Based Hydrogels

#### 2.2.1. Fibrin-ECM Acellular Hydrogels Remain Stable in Culture over 21 Days

**Figure 2:**
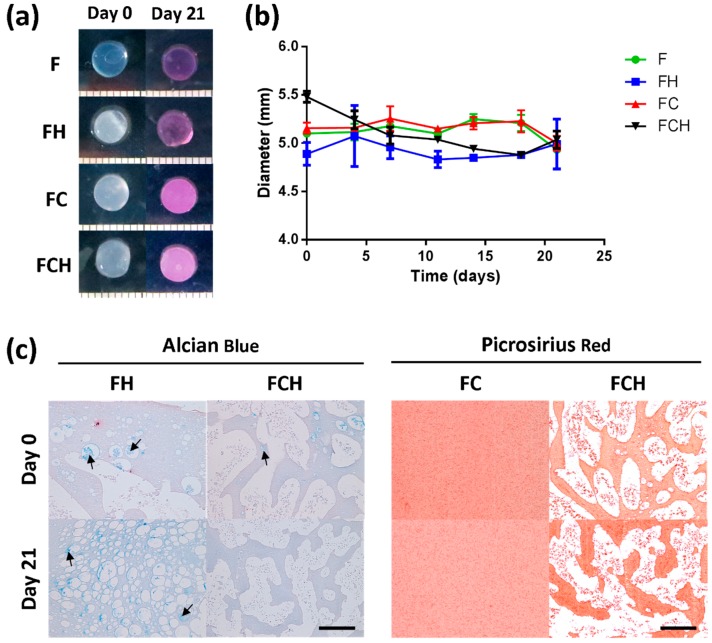
Assessment of geometry and composition stability for fibrin (F, with c_F_ = 50 mg/mL), fibrin–hyaluronic acid (FH with c_F_ = 50 mg/mL and c_H_ = 1.5 mg/mL), fibrin–collagen (FC with c_F_ = mg/mL and c_C_ = 1.33 mg/mL), and fibrin–collagen–hyaluronic acid (FCH with c_F_ = 50 mg/mL, c_C_ = 1.33 mg/mL and c_H_ = 1.5 mg/mL) acellular hydrogels over 21 days. (**a**) Macroscopic images of different gel formulations at day 0 and day 21; (**b**) Determination of diameter of hydrogel constructs; (**c**) Histological assessment of incorporated extracellular matrix (ECM) components of hyaluronic acid (**Left**) and collagen (**Right**). Scale bar = 200 µm.

To assess whether incorporation of ECM components was retained after encapsulation, acellular hydrogels were evaluated over 21 days. All hydrogel formulations maintained their shape (Figure 2a) and initial size of 5 mm (± 0.2) (Figure 2b). Histological assessment for hyaluronic acid (using Alcian blue at pH 2.5) and collagen in different gel formulations revealed a stable amount of HA in Fibrin-hyaluronic acid (FH) groups after 21 days (arrows indicating pockets of HA). However, no HA was detected in fibrin–collagen–hyaluronic acid (FCH) after 21 days (Figure 2c left). Picrosirius red staining of fibrin–collagen (FC) and FCH gels confirmed the incorporation and maintenance of collagen inside the gel over the entire culture period. Small fibres of collagen were observed to be homogeneously distributed within FC gels and in between HA pockets in FCH gels (Figure 2c right).

#### 2.2.2. Fibrin-Based Hydrogels Incorporating Hyaluronic Acid Promote Chondrocyte Proliferation with Enhanced Cell Viability and Increased Cell Spreading

**Figure 3:**
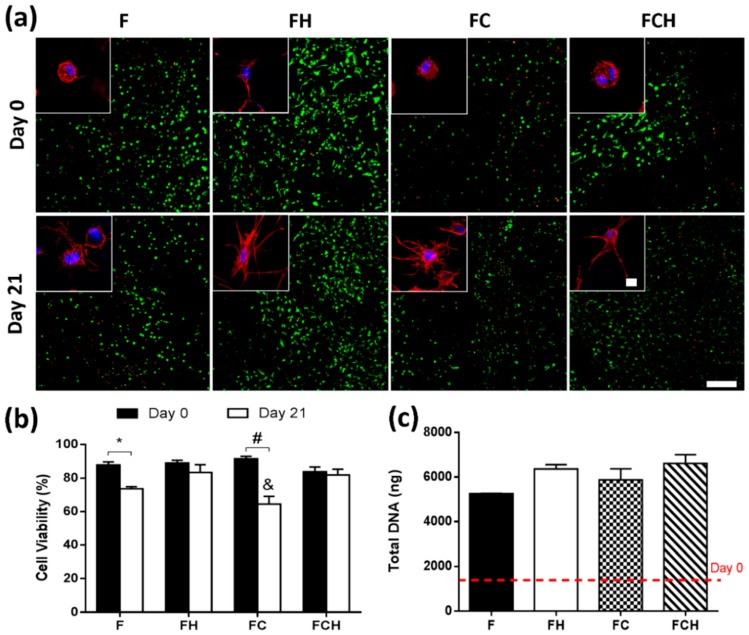
Cell viability, morphology and proliferation in fibrin (F, with c_F_ = 50 mg/mL), fibrin–hyaluronic acid (FH with c_F_ = 50 mg/mL and c_H_ = 1.5 mg/mL), fibrin–collagen (FC with c_F_ = mg/mL and c_C_ = 1.33 mg/mL), and fibrin–collagen–hyaluronic acid (FCH with c_F_ = 50 mg/mL, c_C_ = 1.33 mg/mL and c_H_ = 1.5 mg/mL) hydrogels over 21 days. (**a**) Live/dead imaging of different groups at day 0 (top row) and day 21 (bottom row). Scale bar = 200 µm with cell morphology (inset) for different hydrogel compositions; Scale bar = 10 µm; (**b**) Semi-quantitative analysis of cell viability (%). * (*p* < 0.01), # (*p* < 0.0001); & (*p* < 0.001) indicates statistical significance to day 21 values of FH and FCH; (**c**) Total DNA content (ng). Dashed line indicates day 0 levels.

Cell viability and proliferation was investigated using a combination of semi-quantitative confocal image analysis and biochemical quantification of DNA content. From confocal images of live/dead staining (Figure 3a), cell viability was determined to be 88.3% (±4.6) with no significant difference between hydrogel formulations (Figure 3b). However, after 21 days of cell culture, reduced cell viability was observed in F and FC hydrogels compared to groups which contained hyaluronic acid (FH and FCH). DNA content increased from day 0 levels for all hydrogel formulations, with a four-fold increase observed for fibrin-only hydrogels and a five-fold increase in hydrogels containing hyaluronic acid (Figure 3c).

Cell morphology was investigated using fluorescence staining of actin with phalloidin and nuclei staining with DAPI. Results revealed that incorporating hyaluronic acid promoted cell spreading immediately after cell encapsulation into the material (Figure 3a—top). However, when combined with collagen, cells did not spread as rapidly, and their appearance more closely resembled hydrogels without hyaluronic acid incorporation. After 21 days, cells in all hydrogel formulations exhibited a spread morphology (Figure 3a—bottom) with the least degree of spreading observed in fibrin-only (F) hydrogels compared to all other groups containing ECM components (FH, FC, FCH).

#### 2.2.3. Hyaluronic Acid Enhances sGAG Accumulation and Increases sGAG/Collagen Ratio

**Figure 4:**
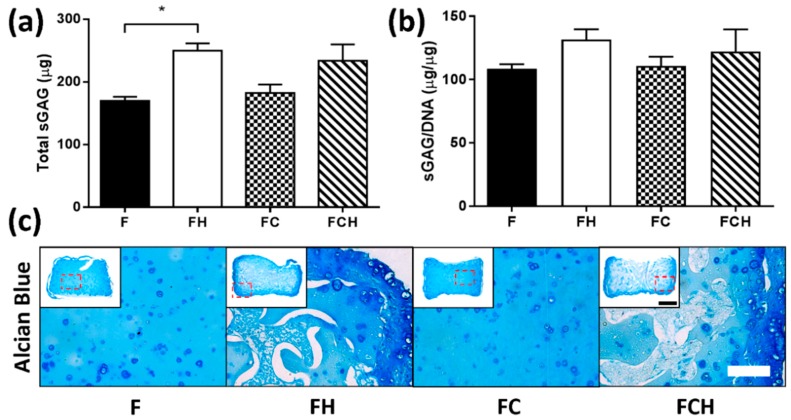
sGAG accumulation in fibrin (F, with c_F_ = 50 mg/mL), fibrin–hyaluronic acid (FH with c_F_ = 50 mg/mL and c_H_ = 1.5 mg/mL), fibrin–collagen (FC with c_F_ = mg/mL and c_C_ = 1.33 mg/mL), and fibrin–collagen–hyaluronic acid (FCH with c_F_ = 50 mg/mL, c_C_ = 1.33 mg/mL and c_H_ = 1.5 mg/mL) hydrogels over 21 days. (**a**) Total sGAG (µg); * indicates statistical significance (*p* < 0.01); (**b**) sGAG/DNA (µg/µg); (**c**) Histological staining using alcian blue for sGAG. Scale bar = 200 µm, inset scale bar = 1 mm.

Matrix deposition was analysed through biochemical analysis and histological staining for sGAG and collagen. Total sGAG accumulation was higher in both FH (250.2 µg ± 23.5) and FCH (233.8 µg ± 45.6) hydrogels compared to materials without HA incorporation (169.9 µg ± 12.7 in F group and 182.4 µg ± 19.4 in FC group), (Figure 4a). On a per cell basis, this trend was maintained, although no statistical difference was observed (Figure 4b). More intense staining for sGAG was observed in the periphery of constructs containing hyaluronic acid, while both F and FC hydrogels exhibited more homogenous staining (Figure 4c).

**Figure 5:**
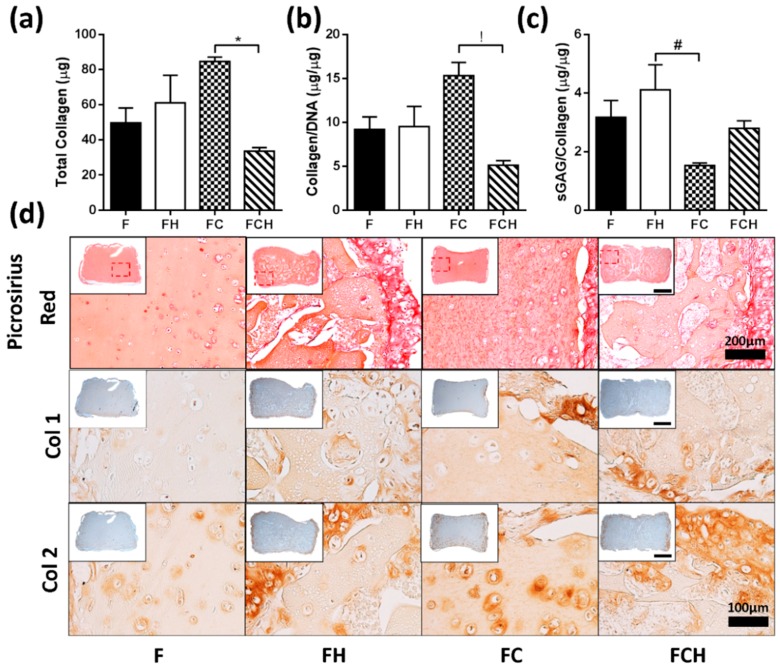
Collagen accumulation in fibrin (F, with c_F_ = 50 mg/mL), fibrin–hyaluronic acid (FH with c_F_ = 50 mg/mL and c_H_ = 1.5 mg/mL), fibrin–collagen (FC with c_F_ = mg/mL and c_C_ = 1.33 mg/mL), and fibrin–collagen–hyaluronic acid (FCH with c_F_ = 50 mg/mL, c_C_ = 1.33 mg/mL and c_H_ = 1.5 mg/mL) hydrogels over 21 days. (**a**) Total (net) Collagen (μg); * indicates significant difference (*p* < 0.05); (**b**) Total (net) collagen/DNA (µg/µg), ! indicates significant difference (*p* < 0.01). For both FC and FCH hydrogels, the initial amount of collagen was subtracted from day 21 values; (**c**) sGAG/total collagen ratio (µg/µg), calculated based on the total collagen (including baseline addition) contained in hydrogel constructs, # indicates significant difference (*p* < 0.05); (**d**) Histological staining with picrosirius red staining for collagen, scale bar = 200 µm, inset scale bar = 1 mm and IHC staining for Col 1 and Col 2, scalebar = 100 µm, inset scale bar = 1 mm.

The highest amount of collagen was accumulated by cells encapsulated in an FC double matrix, and found to be higher compared to the FCH triple matrix (initial amount of collagen was subtracted from day 21 values) (Figure 5a). On a per cell basis, a similar result was observed (Figure 5b). The highest sGAG/collagen ratio was found in FH hydrogels (Figure 5c), which was calculated based on the total collagen (including baseline addition) contained in hydrogel constructs. From histological analysis, collagen deposition in F hydrogels was more homogenous, with higher peripheral collagen matrix observed in FH, FC, and FCH constructs (Figure 5d). Immunohistochemical staining revealed elevated amounts of collagen type I (Col1) in the periphery of FC hydrogels, whereas collagen type II (Col2) was found in the periphery of constructs containing HA and in the pericellular matrix of chondrocytes within FC hydrogels (Figure 5d).

### 2.3. Stage 3—Effect of Increasing Hyaluronic Acid Concentration in Fibrin-Based Hydrogels

#### 2.3.1. Increasing Concentrations of Hyaluronic Acid Do Not Appear to Enhance Cell Viability or Proliferation

**Figure 6:**
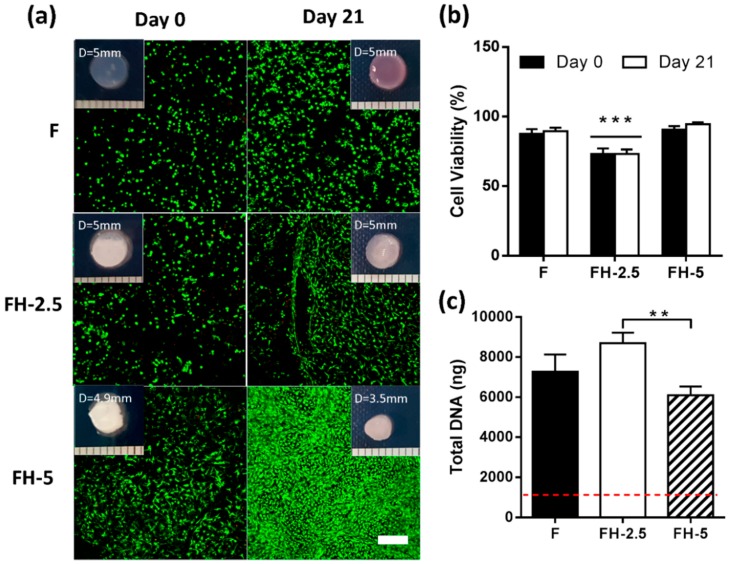
Effect of increasing concentration of hyaluronic acid (0, 2.5, and 5 mg/mL) on cell viability and proliferation in fibrin-based hydrogels over 21 days (**a**) Live/dead imaging at day 0 (left) and day 21 (right). Increased cell number was observed in groups with 5 mg/mL HA concentration. Scale bar = 200 μm; (**b**) Semi-quantitative analysis of cell viability (%). *** indicates statistical significance to all other groups (*p* < 0.0001); (**c**) Total DNA content (ng). ** indicates statistical significance (*p* < 0.01).

Live/dead images were captured at day 0 and day 21, showing good viability at the onset of culture, with increasing density of green fluorescent cells in FH-5 group after three weeks of culture This group also exhibited the highest degree of contraction after 21 days (27.8% ± 3.7%) compared to both FH-2.5 (3.5% ± 3.1%) and F (3.3% ± 3.7%) groups (Figure 6a). Semi quantitative image analysis revealed initial cell viabilities at day 0 of 87.7% ± 3.3 in F hydrogels, with the lowest viability observed for FH-2.5 hydrogels (75.8% ± 7.3) (Figure 6b). No significant changes in cell viability were observed over 21 days for any of the hydrogel formulations investigated. All groups exhibited an increase in DNA content (7-fold, 9.5-fold, and 5-fold increase in F, FH-2.5, and FH-5 respectively) relative to day 0 levels, with a significant difference between FH-2.5 and FH-5, showing a negative effect on cell proliferation with increasing HA concentration (Figure 6c).

#### 2.3.2. Hyaluronic Acid Enhances sGAG and Collagen Deposition in the Periphery of Constructs

**Figure 7:**
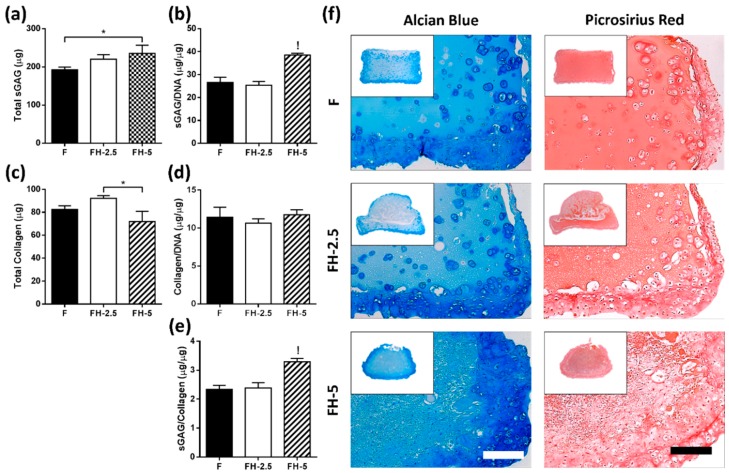
Effect of increasing concentration of hyaluronic acid (0, 2.5, and 5 mg/mL) on matrix accumulation of chondrocytes after 21 days of culture. (**a**) Total sGAG (μg) * indicates statistical difference (*p* < 0.05); (**b**) sGAG/DNA (µg/µg), ! indicates statistical difference compared to all other groups (*p* < 0.001); (**c**) Total Collagen (μg); * indicates significant difference (*p* < 0.05); (**d**) Collagen/DNA (µg/µg) (**e**) sGAG/Collagen ratio (µg/µg); ! indicates significant difference compared to all other groups (*p* < 0.001); (**f**) Histological staining using alcian blue for sGAG and picrosirius red for collagen. Scale bar = 200 µm.

There was an increase in total sGAG when increasing the concentration of incorporated HA with significantly higher levels of sGAG on a per cell basis with the highest amount of HA (FH-5), compared to all other groups (*p* < 0.001) (Figure 7a,b). In addition, a significant difference was observed for total collagen between FH-2.5 and FH-5 (*p* < 0.05). However, no significant differences were detected between groups on a per cell basis when normalising the total collagen to DNA content (Figure 7c,d). The highest ratio of sGAG/collagen was found for the highest FH matrix (3.3 ± 0.1) compared to all other groups (*p* < 0.001) (Figure 7e).

Histology revealed higher sGAG content in the periphery of constructs containing hyaluronic acid (Figure 7f—left). Of note, the staining in FH-5 hydrogels appears more homogeneously distributed, perhaps due to the contraction of constructs. On evaluation of picrosirius red staining, revealing collagen content, more profound staining was observed at the periphery of constructs after 21 days in all groups, similar to alcian blue staining (Figure 7f).

## 3. Discussion

Various cell types have been successfully encapsulated into fibrin hydrogels, such as chondrocytes, stem cells, and fibroblasts [19,20,30,39,40,41]. Fibrin is a viscoelastic material that has been used in different applications, such as a sealant and an adhesive in surgery for wound healing [26,27,28]. However, further improvement can be made using ECM molecules incorporated into the fibrin matrix. It has been shown that ECM molecules, such as collagen and hyaluronic acid, have beneficial effects by promoting proliferation and matrix synthesis [42,43,44]. The overall aim of this work was, therefore, to identify a suitable composition of fibrin hydrogel to promote NP-like matrix formation, which is compatible with commercially available ventures. Fibrin concentration effects were first investigated, followed by determining the influence of ECM molecules, such as hyaluronic acid (HA) and collagen type 1 (Col1), on chondrocyte performance.

### 3.1. Lower Fibrinogen Concentration Results in Hydrogel Contraction

Lower fibrin concentrations (12.5 mg/mL) resulted in significant contraction, which was minimised for concentrations at or above 25 mg/mL. Similar observations have been reported by Eyrich et al., who found that a final fibrin concentration of 25 mg/mL or higher, a calcium concentration of 20 mM and pH between 6.8 and 9 produced a stable, transparent gel for up to three weeks [19]. The contraction of lower fibrin concentrations could be due to the decreased fibre density, and therefore, different pore sizes between the fibres. At higher concentrations, the increasing fibre density leads to stiffer hydrogels, supporting the long-term stability of the hydrogel [45,46]. Kotlarchyk et al. used fluorescently labelled fibrin gels to analyse pore size, and found smaller pore sizes for higher concentrations of fibrin [45]. Another study by Chiu et al. also found a correlation between smaller pore size in higher fibrinogen concentration gels with reduced diffusion and permeability through these gels [47]. These observations correlate with the findings of this work, and perhaps explain the lack of contraction with higher concentrations of fibrinogen, due to the presence of smaller pore sizes between the fibres which are more stable over time.

### 3.2. Higher Fibrin Concentrations Enhance Proliferation

Cell proliferation was found to be enhanced for increasing fibrinogen concentrations. Previous work has reported reduced permeability with increasing concentration, which would be indicative of a diminished nutrient supply for encapsulated cells [47], and may limit cell proliferation. For MSCs, Ho et al. observed increased proliferation with decreasing fibrinogen concentration [40,47]. These differences in proliferation kinetics could be due to the specific cell types utilised. Chondrocytes and MSCs are derived from different niches, and have specific requirements in terms of nutrients such as glucose and oxygen. The microenvironmental niche of chondrocytes comprises of low oxygen and low nutrient supply, due to it being an avascular tissue.

For lower fibrin concentrations, increased cell death was observed, which could be a result of the increased contraction due to lower fibre density [45]. Being softer gels, with larger pores, facilitates cells to exert a force on their surrounding matrix, resulting in contraction of hydrogels and smaller effective pore sizes. These smaller pores, and a higher cell density, may impact on nutrient availability, causing deprivation in the centre, where more cell death occurs.

### 3.3. Hyaluronic Acid Supports Cell Spreading, Proliferation, and the Accumulation of Proteoglycan-Rich Matrix

The enhanced proliferation of chondrocytes in fibrin gels may also be due to increased cell–matrix interaction of fibrin with CD44 surface receptors [48,49]. Cell–matrix interaction occurs through different surface proteins, and it is widely accepted that HA has a high affinity to the cell surface receptor CD44, which is present on different cell types such as chondrocytes, MSCs, and hemopoietic stem cells [50,51,52,53,54]. Once CD44 is activated, an upregulation of the receptor occurs, resulting in reorganisation of the cytoskeleton proteins, clustering of CD44, covalent dimerisation, and binding of HA [55]. High molecular weight (HMW) HAs are long molecules, which can bind to several CD44 receptors at a time, resulting in increased affinity. This binding can affect alignment of intracellular actin filaments, and thus influence the cell shape of chondrocytes [56,57,58], as observed in this work. It is also known that HA mediates both cell–cell and cell–matrix interactions [50,51]. Chondrocytes have been shown to maintain their phenotype when cultured on hyaluronic acid compared to collagen type I hydrogels, with enhanced proliferation and higher expression of chondrogenic markers such as Col2 and aggrecan [59], illustrating the important role of ECM-derived molecules in regulating cellular phenotype. This is in agreement with our findings, whereby enhanced cell proliferation, sGAG, and collagen type II accumulation was observed when hyaluronic acid was incorporated into the fibrin matrix.

### 3.4. Collagen Accumulation Is Enhanced in Fibrin–Collagen Hydrogels

Incorporation of collagen into fibrin-based hydrogels enhanced total collagen accumulation after three weeks in culture, compared to all other groups. Specifically, we observed enhanced col1 deposition at the periphery of hydrogels and enhanced levels of collagen type II within the pericellular matrix (PCM) of chondrocytes. It has been shown that fibrin can interact with CD44 and the integrin αvβ3. αvβ3 is an integrin which has no intracellular effect on chondrocytes when present or knocked out, indicating no major role when attachment of chondrocytes occurs [60]. The affinity to the integrin and the cell–matrix attachment using the RGD motifs of fibrin may be higher than the interaction with CD44, which explains the limited effect of fibrin gel on chondrocytes. Fibrin provides binding sites, which do not affect chondrocytes further through intracellular signalling. Previous work has shown higher levels of col2 expression of NP cells in fibrin–collagen hydrogels both in vitro and in vivo [20]. It has also been shown that collagen type I can specifically bind to fibrin, using the integrin αvβ3 connection as a functional interface matrix which could activate different intracellular pathways [61]. Further, it has been shown that the combination of collagen and fibrin alters the mechanical properties [62,63], which could also be responsible for phenotypic changes.

### 3.5. Hyaluronic Acid Appears to Supress Collagen ECM Accumulation

Incorporation of hyaluronic acid into fibrin–collagen matrices appeared to suppress levels of total collagen and increased levels of sGAG, possibly due to the activation of CD44 receptors. Collagen–cell interactions mainly activate pathways involved with cell adhesion, cell migration, and stress fibre assembly in AF and NP cells, which are not primarily involved in matrix deposition [64]. Mahapatra et al., examined the behaviour of chondrocytes in a triple matrix consisting of alginate, collagen, and HA (Alg–Col–HA), compared to a double matrix of alginate and HA only (Alg–HA) [65]. The authors found that the triple matrix supported proliferation significantly more than the double matrix after 21 days, with enhanced expression of chondrogenic markers. This contrasts with our findings, where the double matrix with hyaluronic acid and the triple matrix appear to have similar chondrogenic capacities. However, it should be noted that Mahapatra et al. utilised alginate as the supporting gel, which does not provide binding sites, and may explain the different results observed.

### 3.6. Incorporation of 5 mg/mL HMW Hyaluronic Acid into Fibrin Matrices Enhances sGAG/Collagen Ratio

In the final stage of this study, the effect of different amounts of hyaluronic incorporated into the fibrin matrix was investigated. Previous work has investigated the effect of different concentrations of HA (0.1–3.0 mg/mL) on chondrocytes in alginate hydrogels [66]. The authors observed a trend towards higher levels of DNA, sGAG, and collagen after 14 days with lower concentrations (0.1–1 mg/mL) of HA. However, at higher concentrations, diminished DNA and collagen contents were observed, similar to the present study. It has been shown HMW HA, as used in this work, can bind to several CD44 surface markers at a time [57,58], indicating that saturation of CD44–HA interaction can occur even with a lower concentration of HMW HA molecules. This suggests a threshold exists beyond which increasing HA concentration will have no additional benefit.

Another possible explanation for the observed enhancement of sGAG with higher concentrations of HA is due to a physical change in hydrogel network, rather than a cellular activation alone. HA is inherently difficult to retain in hydrogel systems, and easily diffuses into the surrounding media [67], which was also evidenced in acellular hydrogel studies in the present work. Therefore, it is difficult to determine for what period lower HA concentrations are effective to elicit a beneficial response. At the highest HA concentration (FH-5), shrinkage of constructs was observed, which may have led to entrapment of HA within the fibrin matrix network, resulting in a higher degree of cell–cell contact and cellular condensation, thereby promoting chondrogenesis. Whether the beneficial effect of the highest concentration of HA is due to the binding of HA or due to the initiated condensation of the construct remains unclear, and warrants further investigation. Overall, these findings suggest, despite increased contraction, a concentration of 5 mg/mL HA to be suitable for disc repair, due to the enhanced NP-like matrix accumulation of articular chondrocytes within this network.

## 4. Materials and Methods

### 4.1. Study Design

**Figure 8:**
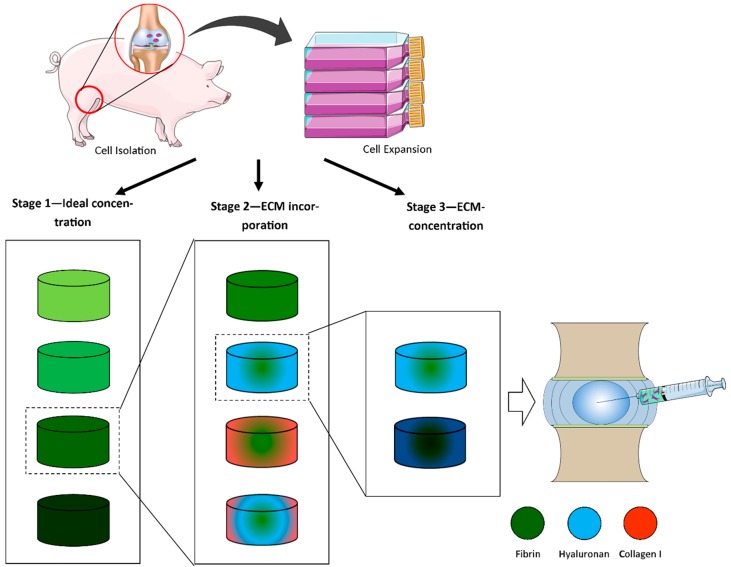
Schematic of experimental design of the fibrin study. In stage 1, different final fibrin concentrations (12.5, 25, 37.5, and 50 mg/mL) were investigated. Stage 2 involved incorporation of collagen and hyaluronic acid into fibrin. Stage 3 examined the influence of increasing HA concentrations (2.5, 5 mg/mL) on promoting matrix accumulation by articular-derived chondrocytes.

This study consisted of three different stages (Figure 8). In all stages, articular chondrocytes were isolated and expanded in monolayer from porcine knee joints until passage 1 or 2. In stage 1, different final fibrin concentrations (12.5, 25, 37.5, and 50 mg/mL) were investigated. Stage 2 involved incorporation of collagen and hyaluronic acid into fibrin. Stage 3 examined the influence of increasing HA concentration on promoting matrix accumulation by articular-derived chondrocytes.

### 4.2. Hydrogel Fabrication

The following components were used for the fabrication of different hydrogel compositions: fibrinogen type I-S (60–85% protein, ~10% sodium citrate, and ~15% sodium chloride, Sigma-Aldrich F8630, Arklow, Ireland), thrombin from bovine plasma (Sigma-Aldrich, T4648, Arklow, Ireland), hyaluronic acid sodium salt from *Streptococcus equi* (MW = 1.5 − 1.8 × 10^6^ Da, Sigma-Aldrich 53747, Arklow, Ireland), and collagen type I, rat tail high concentration (Corning™ 354249, Corning, NY, USA).

#### 4.2.1. Preparation of Fibrin Hydrogels with Various Concentrations of Fibrinogen

Fibrin hydrogels were produced by solubilising desired concentrations of fibrinogen type I-S (60–85% protein, ~10% sodium citrate, and ~15% sodium chloride, Sigma-Aldrich, Arklow, Ireland) in 10,000 KIU/mL aprotinin solution (Nordic Pharma, Limhamn, Sweden) containing 19 mg/mL sodium chloride at 37 °C, and crosslinked using a pre-warmed thrombin solution (5 U/mL in 40 mM CaCl_2_, pH 7) and allowed to gel (see Table 1). To allow gelation to proceed, hydrogels were incubated for 30 min at 37 °C in a humidified atmosphere.

#### 4.2.2. Fibrin Hydrogels Containing ECM Components (Collagen, Hyaluronic Acid)

Specific fibrin-based hydrogels (fibrin only, fibrin–hyaluronic acid, fibrin–collagen, and fibrin–collagen–hyaluronic acid) were fabricated at 37 °C, and allowed to crosslink for 60 min in a humidified atmosphere inside a 3% agarose mould (see Table 2). To create fibrin–hyaluronic acid hydrogels, hyaluronic acid (3 mg/mL) was dissolved in thrombin and combined with fibrinogen to yield a final concentration containing 1.5 mg/mL hyaluronic acid. To create fibrin–collagen gels, collagen type I (Corning, Corning, NY, USA) was incorporated at a final concentration of 1.33 mg/mL into a fibrinogen/thrombin mixture as follows: soluble collagen with an initial concentration of 6 mg/mL in 0.02 M acetic acid was neutralised using a buffer containing NaOH, NaHCO_3_, HEPES, and 10× RPMI media, and combined with low-glucose Dulbecco’s modified Eagle’s medium (1000 mg/mL d-glucose, Sigma, Arklow, Ireland). Thrombin (5 U/mL) was added and crosslinked using pre-warmed fibrinogen at a specified concentration to obtain a final concentration of 50 mg/mL fibrinogen. For fibrin–collagen–hyaluronic hydrogels, a thrombin–hyaluronic acid mix (5.2 mg/mL initial HA) was combined with neutralised collagen in a similar fashion as described for fibrin–collagen hydrogels, and crosslinked using pre-warmed fibrinogen.

#### 4.2.3. Fibrin-Based Hydrogels with Increasing Hyaluronic Acid Concentrations

Fibrin–hyaluronic acid hydrogels were created as described in the previous Section 4.2.2 using an initial HA concentration of 5 mg/mL in thrombin (5 U/mL). Thrombin–HA solution was combined with fibrinogen (100 mg/mL) with and without the addition of 5 mg/mL HA at a ratio of 1:1 to obtain the desired final concentrations of 2.5 mg/mL or 5 mg/mL, respectively. Cells were combined with the fibrinogen solution prior to incorporation of thrombin. To ensure crosslinking, all hydrogels were incubated for 60 min at 37 °C in a humidified atmosphere.

### 4.3. Chondrocyte Isolation and Monolayer Expansion

Juvenile porcine chondrocytes (pCC) were isolated from articular cartilage of 4-month-old female porcine donors (~50 kg). Tissue was finely minced and digested in collagenase for 12 h at 37 °C. Digested tissue/cell suspensions were passed through a 40 µm cell strainer to remove tissue debris, and washed three times by repeated centrifugation at 650 g for 5 min. Cell viability was determined with a haemocytometer and trypan blue exclusion. Cells were seeded at an initial density of 5 × 10^3^ cells/cm^2^ in media consisting of low-glucose Dulbecco’s modified Eagle’s medium (LG-DMEM; 1 mg/mL d-glucose, Sigma) supplemented with 10% foetal bovine serum (FBS), penicillin (100 U/mL)–streptomycin (100 µg/mL) (all GIBCO, Invitrogen, Dublin, Ireland), and amphotericin B (0.25 µg/mL, Sigma-Aldrich, Arklow, Ireland) at 37 °C and 5% CO_2_, and cultured until passage 1 or 2.

### 4.4. Cell Encapsulation

#### 4.4.1. Varied Fibrin Concentration Hydrogels

For fibrin encapsulation, pCCs were suspended in fibrinogen solutions (Table 1) at a cell density of 8 × 10^6^ cells/mL. This was combined with thrombin solution (1:1 ratio) and allowed to gel in an 3% agarose mould, pre-soaked in cell culture media to ensure nutrient supply for cells for 30 min at 37 °C to produce cylindrical constructs (Ø5 mm × 3 mm thickness), and a final cell seeding density of 4 × 10^6^ cells/mL.

#### 4.4.2. Fibrin–Collagen and Fibrin–Hyaluronic Acid–Collagen Hydrogels

For encapsulation, pCCs were suspended in pre-warmed fibrinogen solution and combined with collagen–thrombin or collagen–HA–thrombin solution, respectively, to obtain a final cell seeding density of 4 × 10^6^ cells/mL at matrix concentrations, described in Table 2 Gelling took place in 3% agarose moulds soaked in media for 60 min at 37 °C, producing cylindrical constructs (Ø5 mm × 3 mm thickness).

#### 4.4.3. Fibrin–Hyaluronic Acid Hydrogels

For encapsulation of pCCs into different concentrations of fibrin–hyaluronic acid hydrogels, cells were suspended in fibrinogen solution (100 mg/mL) with or without incorporation of 5 mg/mL HA. This was combined at a ratio of 1:1 with 5 U/mL thrombin–HA solution, to obtain a final cell seeding density of 4 × 10^6^ cells/mL, and allowed to gel in a 3% agarose mould pre-soaked in media for 60 min at 37 °C to produce cylindrical constructs (Ø5 mm × 3 mm thickness). All samples were allowed to equilibrate overnight before initiation of experiments.

### 4.5. Culture of Chondrocyte Laden Fibrin-Based Constructs

All constructs were maintained at 37 °C and 5% oxygen conditions in 2 mL of chemically defined medium (CDM) consisting of lgDMEM supplemented with penicillin (100 U/mL)–streptomycin (100 µg/mL) (both GIBCO, Biosciences, Ireland), 40 µg/mL L-proline, 50 µg/mL L-ascorbic acid-2-phosphate, 1.5 mg/mL BSA, 1× insulin–transferrin–selenium, 100 nM dexamethasone (all from Sigma–Aldrich, Ireland), and 5% FBS. Medium was exchanged twice weekly and sampled for biochemical analysis for a total duration of 21 days.

### 4.6. Determination of Hydrogel Contraction

Hydrogel contraction kinetics were determined via image analysis using a digital camera (Canon Powershot SX240HS) at each feeding period (twice weekly). The average diameter of three constructs was determined using image analysis software (ImageJ, National Institutes of Health, and Bethesda, Maryland).

### 4.7. Live/Dead Analysis

Cell viability was assessed using a LIVE/DEAD^®^ Viability/Cytotoxicity Assay Kit (Invitrogen). Briefly, hydrogels were cut in half and washed in phosphate buffered saline (PBS, Sigma, Arklow, Ireland), followed by incubation in PBS containing 2 μM calcein AM (live cell membrane, abs/em = 494/517 nm) and 4 μM ethidium homodimer-1 (dead cell DNA, ex/em = 528/617 nm; both from Cambridge Bioscience, Cambridge, UK). Hydrogels were again washed in PBS, imaged with a Leica SP8 scanning confocal microscope at 515 and 615 nm and assessed using Leica Application Suit X (LAS X) Software.

### 4.8. Cell Shape Analysis

Cell shape was analysed on fixed samples (4% PFA, Sigma-Aldrich, Arklow, Ireland). After fixation, samples were thoroughly washed in PBS and permeabilised using 0.5% Triton-X. Fluorescent stains were diluted in 1.5% BSA solution (for rhodamine phalloidin (ex/em = 540/565 nm) at 1:40, and for DAPI (ex/em = 364/454 nm), a 1:1000 dilution was used). Stains were incubated for 1 h (rhodamine phalloidin) and 10 min (DAPI) at room temperature, avoiding artificial light, imaged with a Leica SP8 scanning confocal at 358/524 and 540/565 nm channels, and assessed using Leica Application Suit X (LAS X) Software.

### 4.9. Quantitative Biochemical Analysis

To quantify the accumulation of biochemical constituents, samples were digested with 125 μg/mL papain in 0.1 M sodium acetate, 5 mM L-cysteine-HCl, 0.05 M EDTA, pH 6.0 (all from Sigma-Aldrich) at 60 °C under constant rotation for 18 h (*n* = 3 for each group). DNA content of each sample was quantified using the Hoechst bisbenzimide 33258 dye assay, with a calf thymus DNA standard. sGAG content was quantified using the dimethylmethylene blue dye-binding assay (Blyscan, Biocolor Ltd., Carrick Fergus, Northern Ireland), with a chondroitin sulphate standard. Total collagen content was determined by measuring the hydroxyproline content. Samples were hydrolysed at 110 °C for 18 h in 38% HCl, and assayed using a chloramine-T assay [68] with a hydroxyproline/collagen ratio of 1:7.69 [69]. Each biochemical constituent was normalised to DNA content.

### 4.10. Histology

At each time point, samples were fixed in 4% paraformaldehyde overnight at 4 °C, followed by repeated washings in PBS. Fixed samples were dehydrated in a graded series of ethanol (70% to 100%), embedded in paraffin wax, sectioned at 7 μm, and affixed to microscope slides. Sections were stained with 1% alcian blue 8GX in 0.1 M HCL to assess sGAG content and picrosirius red to assess collagen distribution (all from Sigma-Aldrich). Collagen types 1 and 2 were evaluated using a standard immunohistochemical technique. Briefly, sections were treated with peroxidase, followed by treatment with chondroitinase ABC (Sigma-Aldrich) in a humidified environment at 37 °C to enhance permeability of the extracellular matrix. Sections were incubated with goat serum to block non-specific sites and collagen type I (abcam 90395, 1:400), collagen type II (abcam 3092, 1:100) primary antibodies (mouse monoclonal, IgG, Cambridge, UK) were applied overnight at 4 °C. Next, the secondary antibody (anti-mouse IgG biotin conjugate, 1.5:200) (Sigma-Aldrich B7151) was added for 1 h, followed by incubation with ABC reagent (Vectastain PK-400, Vector Labs, Peterborough, UK) for 45 min. Finally, sections were developed with DAB peroxidase (Vector Labs) for 5 min. Positive and negative controls were included in the immunohistochemistry staining protocol for each batch.

### 4.11. Statistical Analysis

Statistical analyses were performed using GraphPad Prism (version 6, GraphPad Software, La Jolla, CA, USA) software with 3–4 samples analysed for each experimental group. The sGAG/collagen ratio was determined by dividing sGAG (µg) by collagen (µg). One-way ANOVA was used for analysis of variance with Tukey’s multiple comparisons test to compare between groups. Results are displayed as mean ± standard deviation, with significance accepted at a level of *p* < 0.05.

## 5. Conclusions

In this work, we explored the incorporation of ECM molecules into fibrin-based hydrogels in order to develop a suitable injectable system to facilitate cellular delivery. Significant hydrogel contraction was observed using low (12.5 mg/mL) fibrin concentrations, with limited contraction for concentrations at or above 25 mg/mL. With the incorporation of ECM components, cell viability was seen to be maintained when HA was incorporated. HA also enhanced sGAG accumulation and tended to suppress collagen formation at higher concentrations. There did not appear to be any significant benefit of incorporating collagen in the fibrin matrix. Lastly, we showed that there is an overall increase in the sGAG to collagen ratio when increasing HA concentration in the fibrin matrix to 5 mg/mL. Taken together, these results suggest that incorporation HA can enhance the bioactivity of fibrin-based hydrogels, which may help advance the clinical potential of commercial cell and biomaterial ventures in the treatment of IVD regeneration.

## Figures and Tables

**Table 1:** Preparation of fibrin hydrogels with various concentrations (12.5, 25, 37.5, and 50 mg/mL).

Fibrinogen Concentration (mg/mL)	Fibrinogen: Thrombin Ratio	Final Fibrinogen Concentration (mg/mL)
25	1:1	12.5
50	1:1	25
75	1:1	37.5
100	1:1	50

**Table 2:** Different materials investigated and their final concentrations.

	Abbreviation	Final Fibrin Conc. (mg/mL)	Col Conc. (mg/mL)	Hyaluronic Acid Conc. (mg/mL)
Fibrin	F	50		
Fibrin–HA	FH	50		1.5
Fibrin–COL	FC	50	1.33	
Fibrin–Col–HA	FCH	50	1.33	1.5
